# First-in-human *ex-vivo* validation of carbonic anhydrase IX-PET for high-risk renal cancer (CAT-VHL - PNRR-POC-2023-12377493)

**DOI:** 10.7150/thno.122256

**Published:** 2026-05-18

**Authors:** Alessandro Larcher, Fabrizia Gelardi, Lidija Antunovic, Annarita Savi, Michela Olivieri, Paolo Rainone, Martina Sollini, Rosa Maria Moresco, Chiara Re, Francesco Cei, Federico Belladelli, Giacomo Musso, Lucia Salerno, Francesco De Cobelli, Nazario Tenace, Maurilio Ponzoni, Antonio Cigliola, Paolo Verze, Roberto Bertini, Alberto Briganti, Francesco Montorsi, Umberto Capitanio, Andrea Salonia, Arturo Chiti

**Affiliations:** 1 Department of Urology, Division of Oncology, Urological Research Institute, IRCCS Ospedale San Raffaele, Milan, Italy.; 2 VHL Program, IRCCS Ospedale San Raffaele, Milan, Italy.; 3 Department of Nuclear Medicine, IRCCS Ospedale San Raffaele, Milan, Italy.; 4 Institute of Molecular Bioimaging and Physiology-IBFM, CNR, Segrate, Italy.; 5 Vita-Salute San Raffaele University, Milan, Italy.; 6 Department of Medicine and Surgery, University of Milano-Bicocca, Monza, Italy.; 7 Department of Radiology, IRCCS Ospedale San Raffaele, Milan, Italy.; 8 Department of Pathology, IRCCS Ospedale San Raffaele, Milan, Italy.; 9 Department of Oncology, IRCCS Ospedale San Raffaele, Milan, Italy.; 10 Department of Urology, AOU San Giovanni di Dio Ruggi d'Aragona, Salerno, Italy.; 11 Università degli Studi di Salerno, Salerno, Italy.

**Keywords:** renal cell carcinoma, carbonic anhydrase IX, positron-emission tomography, theranostics, tumor heterogeneity

## Abstract

**Rationale:**

Carbonic Anhydrase IX (CAIX) positron emission tomography (PET) is an accurate and non-invasive imaging modality for the detection and characterisation of clear-cell renal cell carcinoma (ccRCC) but evidence in the setting of high-risk renal cancer is lacking. We conducted an exploratory investigation to integrate whole-body CAIX-PET imaging with tissue-level validation and to assess the potential impact of CAIX-PET on systemic staging in patients diagnosed with high-risk renal cancer.

**Methods:**

Patients with cT3–cT4 or cN1 renal cancer scheduled for surgery underwent PET/CT imaging after intravenous administration of 37 MBq ±10% of [89Zr]Zr-girentuximab. Whole-body *in-vivo* was performed 5 ± 2 days following radiopharmaceutical injection, and surgical resection was planned 14 ± 2 days following radiopharmaceutical injection. Resected specimens were subsequently analyzed ex vivo using dedicated preclinical PET/CT imaging and correlated with histopathology and CAIX immunohistochemistry.

**Results:**

No adverse events following [^89^Zr]Zr-girentuximab administration were recorded and the procedure was deemed non-relevant in terms of radiation exposure for the surgical team. At *in-vivo* imaging, CAIX-PET resulted positive in 2 patients with clear cell renal cell carcinoma and negative in 1 patient with chromophobe renal cell carcinoma. At *ex-vivo* imaging, images overlap allowed for the assessment of spatial co-localization of regions with increased radiopharmaceutical uptake and high expression of CAIX at immunohistochemistry. In 1 patient, focal uptake in the fourth rib at CAIX-PET was confirmed as metastatic ccRCC. In 1 patient, suspicious lymph nodes at standard imaging without PET uptake were negative at final pathology.

**Conclusion:**

Our findings generate the hypothesis that CAIX-PET might yield crucial information on cancer aggressiveness and systemic staging with potential key diagnostic, therapeutic and prognostic implications for patients with high-risk renal cancer.

## Introduction

Clear cell renal cell carcinoma (ccRCC), the most common and most aggressive renal cancer subtype 1, is characterized by substantial biological heterogeneity and unpredictable metastatic behavior 2 . Conventional imaging does not capture spatial molecular variability in the primary and may fail to provide accurate systemic staging in case of metastatic disease.

Carbonic anhydrase IX (CAIX) represents a hallmark of ccRCC biology and a promising target for molecular imaging, currently under scrutiny for the diagnosis of clear cell renal cell carcinoma (ccRCC). The sensitivity and specificity of CAIX-PET were 86% and 87% in a phase 3 clinical trial assessing patients with an indeterminate cT1 renal mass elected for nephrectomy 4. However, no evidence is available in the setting of high-risk renal cancer 5.

During cancer evolution and clonal de-differentiation, proximal-tubule cells might lose CAIX expression 6 either in the primary tumor or in distant metastases; hence preventing detection with CAIX-PET imaging. For this reason, with the intent to provide preliminary clinical data supporting the use of CAIX-PET in high-risk ccRCC, we conducted an exploratory investigation to integrate whole-body CAIX-PET imaging with tissue-level validation and to assess the potential impact of CAIX-PET on systemic staging in patients diagnosed with high-risk renal cancer.

## Materials and Methods

First, we obtained approval by the Ethical Committee for early access program under compassionate use (Comitato Etico Territoriale Lombardia 1 - N°20-2024) and subsequently, we obtained approval by the Ethical Committee and AIFA - Agenzia Italiana del Farmaco for a phase 2 clinical trial (CAT-VHL - PNRR-POC-2023-12377493 trial; https://clinicaltrials.gov/study/NCT07171905).

We selected patients diagnosed with high-risk renal cancer, defined as stage cT3-cT4 or cN1 at conventional imaging and administered a single intravenous dose of the standard diagnostic activity (37 MBq ± 10%) of [^89^Zr]Zr-girentuximab, an anti-CAIX monoclonal antibody radiolabeled with Zirconium-89. Subsequently, all patients were subjected to whole-body imaging with a hybrid PET/CT scanner (GE Healthcare Omni Legend) on day 5 ± 2 after radiopharmaceutical administration. PET/CT scanner characteristics and acquisition parameters are described in *Table S1*.

Following the approval of a dedicated protocol for radioprotection by the institutional physics service, surgery was planned on day 14 ± 2 after radiopharmaceutical injection. This timing was chosen based on theoretical dosimetry estimates and the long half-life of Zirconium-89 (78.4 hours), with the intent to maximally reduce the exposure of the surgical team and at the same time to maximally increase the likelihood of detecting adequate activity in cancer tissue. Immediately after surgery, the renal specimens were initially fixed in formalin and subsequently sectioned along parafrontal planes to obtain slices suitable for the dimensions of the imaging equipment. The slices containing the lesion and adjacent renal parenchyma were then placed in sealed polyethylene specimen bags. Subsequently, we performed ex vivo studies using a preclinical CT and PET scanner (X-cube® and β-cube®, Molecubes, Gent, Belgium 7). Each surgical specimen was placed on the scanner bed and first imaged with the preclinical CT scanner (X-cube®) in high-resolution mode for approximately three minutes. The bed was subsequently transferred to the preclinical PET scanner (β-Cube®) and scanned for a 180-minute acquisition. CT data were reconstructed with an isotropic pixel size of 200 µm using the ISRA algorithm, while PET data were reconstructed using the OSEM algorithm (10 iterations, isotropic voxel size 400 µm), including corrections for tracer decay and attenuation. Following imaging, specimens were processed for histopathological evaluation by an expert genitourinary pathologist.

## Results and Discussion

Patients’ data are described in *Table 1*. No adverse events following [^89^Zr]Zr-girentuximab administration were recorded. Radiation exposure of the surgical team was negligible, with a maximal adsorbed dose of 0.018 mSv measured by cuff dosimeter; accordingly, the procedure was deemed non-relevant in terms of radiation exposure. Moreover, surgery was not technically influenced by exposure to the radiopharmaceutical in any way. In one case, a small accidental pleural opening occurred and required suturing. The event was largely anticipated owing to the location and dimension of the primary tumor. The patient had fever and transient acute respiratory failure treated with antibiotic and oxygen support (Clavien-Dindo 2). No major postoperative complications were recorded.

**Figure 1:**
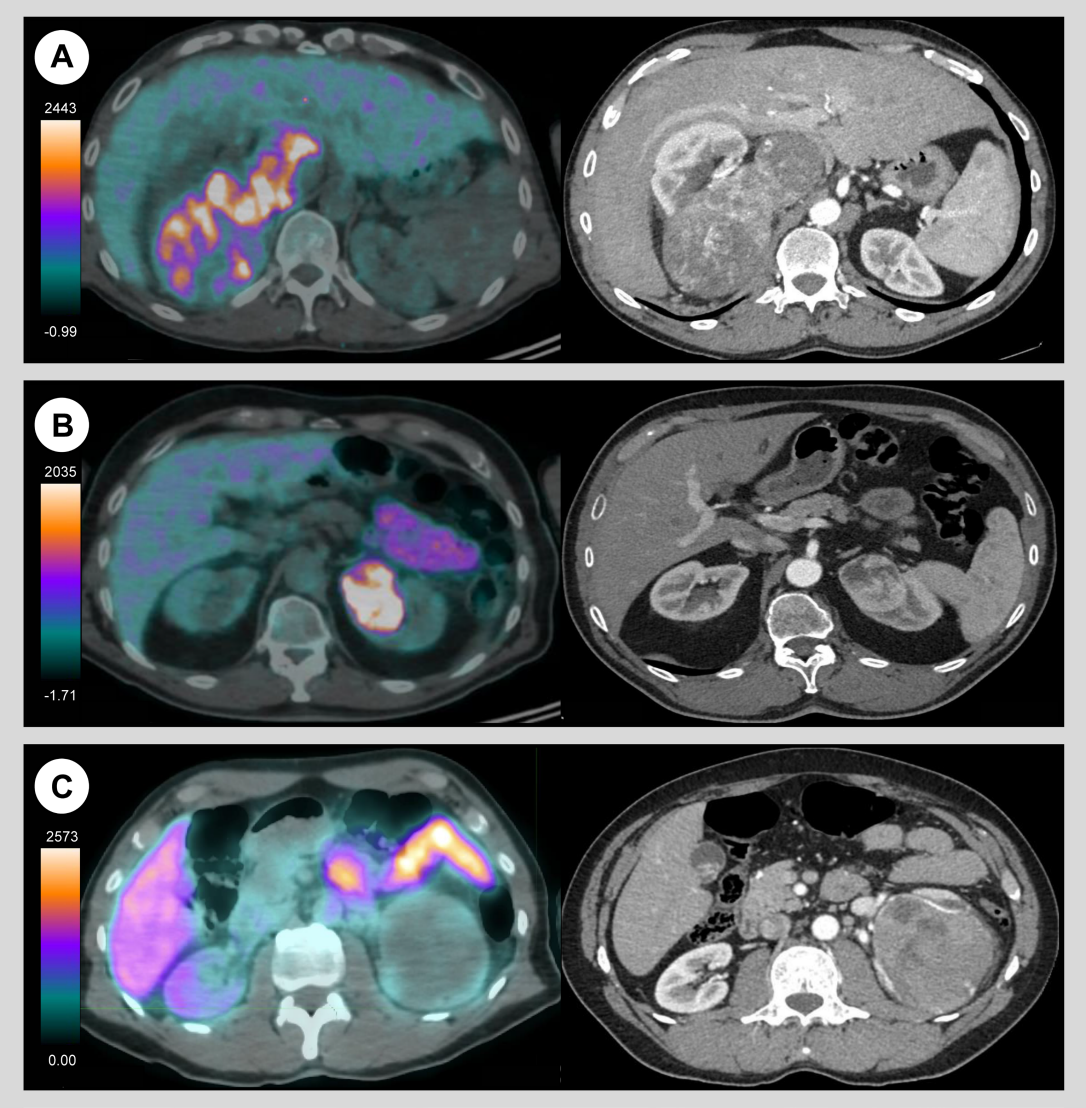
Axial *in-vivo* CAIX-PET/CT (left) and standard contrast-enhanced CT (right) images in three patients with high-risk renal cancer. CAIX-PET demonstrated significant albeit spatially heterogeneous uptake in the primary renal tumor in two cases -right kidney (A) and left kidney (B)- later confirmed as pT3a renal cell carcinoma at final pathology. In one case (C), CAIX-PET was negative, with no significantly detectable tumor uptake. This finding was confirmed at final pathology as a pT3a chromophobe tumor.

CAIX-PET resulted positive in two cases where *in-vivo* studies exhibited intense albeit spatially heterogeneous radiopharmaceutical uptake in primary tumour. Pathology confirmed ccRCC subtype (*Figure 1A and 1B*). Conversely, CAIX-PET resulted negative in a third case, and *in-vivo* study exhibited no uptake in the primary. Pathology revealed chromophobe subtype (*Figure 1C*). All cases were high-risk renal cancer with either high stage and high grade features.

Images overlap allowed for the assessment of spatial co-localization of regions with increased radiopharmaceutical uptake and high expression of CAIX at immunohistochemistry (*Video 1 and Figure S2*). These regions were populated by viable cancer cells with abundant clear cell cytoplasm showing relatively aggressive features such as nucleolar grade 2 and 3 8. Consistently, regions with no radiopharmaceutical uptake were also CAIX negative at immunohistochemistry staining and were consistent with areas of fibrous and acellular myxoid degeneration that are often present in ccRCC. Of note, CAIX staining intensity and distribution was homogeneous in neoplastic areas with different architectural growth pattern but similar morphological features.

**Figure 3:**
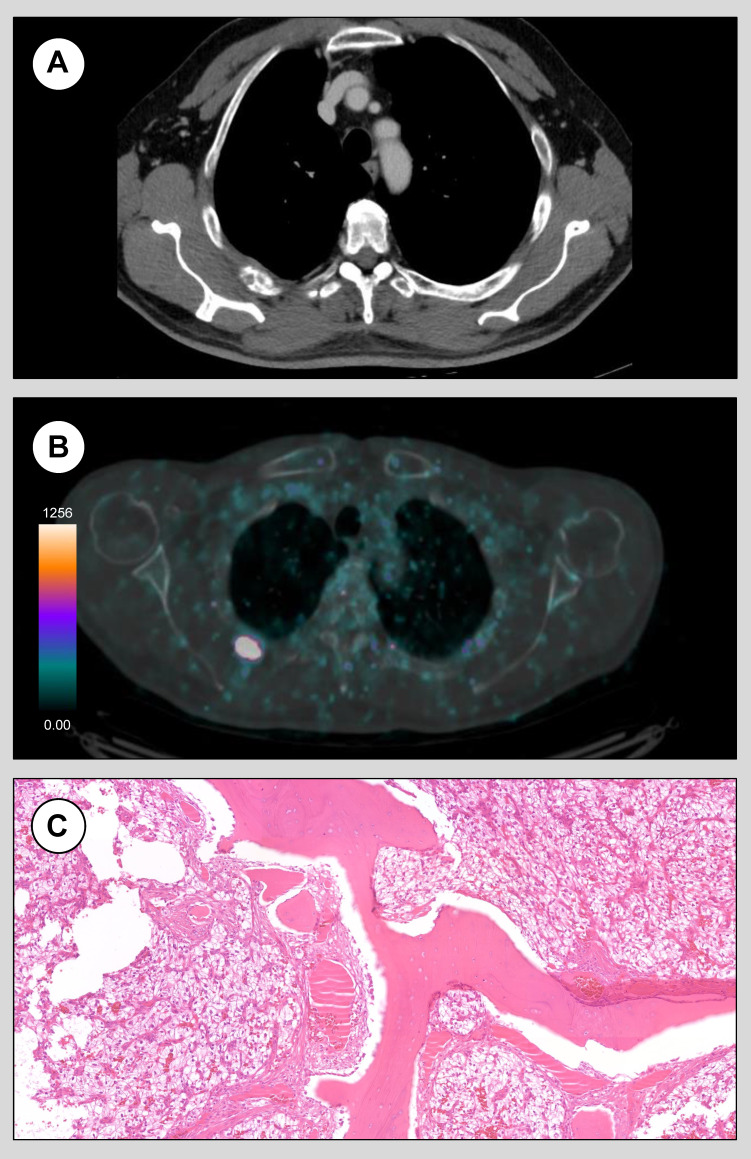
Axial standard chest CT images (A) and CAIX-PET images (B) of case 2. CAIX-PET prompted the reclassification from cM0 to cM1 by detecting pathological radiopharmaceutical uptake in the fourth rib. Following surgical metastasectomy, final pathology confirmed the presence of osseous trabeculae interspersed with richly vascularized neoplastic tissue composed of cells with abundant clear cytoplasm, consistent with metastatic ccRCC.

Whole body *in-vivo* CAIX-PET findings led to a significant revision of the initial staging. A case of cT3a cN0 cM0 renal cancer at standard imaging (*Figure 3A*) has been upstaged from cM0 to cM1 because of a pathological uptake at the fourth rib detected at CAIX-PET (*Figure 3B*) consistent with positive finding in the primary. Following multidisciplinary team discussion, the patient was elected for surgical resection of the rib lesion, and pathology confirmed a ccRCC metastasis (*Figure 3C*). Conversely, a case of cT2b cN1 cM0 renal cancer at standard imaging has been downstaged from cN1 to cN0 because of the absence of any uptake in the retroperitoneal lymph nodes at CAIX-PET despite positive finding in the primary. Final pathology confirmed pN0 disease and no nodal recurrence was detected at 12-months follow-up.

The novelty and uniqueness of these findings deserve special consideration. With respect to intra-tumoral heterogeneity, CAIX-PET can discriminate different regions within the same tumor with distinct and peculiar pathologic features. Our results highlight that the negligible uptake at CAIX-PET in certain portions of the tumor should not be regarded as false-negative outcome owing to sub-optimal radiopharmaceutical pharmacokinetics, but rather as true negative outcome owing to the lack of target expression at cellular level. Of note, false negative results of CAIX-PET are biologically plausible in case of high-risk localized or metastatic ccRCC without CAIX expression, and the frequency of this event cannot be determined based on our limited sample.

These observations might propel novel research lines with respect to relevant unmet clinical needs in the care of ccRCC patients. For instance, renal biopsy fails to provide a diagnosis in 10% of the cases 9, due to sampling of uninformative tissue. In this setting, CAIX-PET image-guided biopsy could reduce the rate of non-diagnostic biopsies by targeting the most informative areas of the tumor. Moreover, pre-treatment CAIX-PET-based estimation of cancer aggressiveness might justify a more demolitive surgical strategy such as radical instead of partial nephrectomy 9, ultimately reducing the risk of recurrence 10 in surgical candidates or might identify areas requiring radiation boost with supplemental dose in comorbid patients elected for external beam radiation therapy 11,12. Finally, in the context of the ongoing development of novel theranostic agents for ccRCC 13,14, the pre-therapeutical identification of portions with limited radiopharmaceutical uptake could have critical implications with respect to dosimetry planning, as these instances have intrinsic risk of undertreatment.

With respect to detection of ccRCC metastases, better strategies to capture oligometastatic disease are critically needed, since most renal cancer metastases are asymptomatic, and fewer than 20% of cM+ renal cancer patients develop signs or symptoms 3. Current international guidelines do not support the use of any nuclear imaging modality for renal staging 9 given the limitations of available radiopharmaceuticals. Specifically, the accuracy of [^18^F]F-deoxyglucose (FDG) is low, and the evidence supporting the use of prostate-specific membrane antigen (PSMA) 15, [^99^TC]TC-sestamibi 16 or [^18^F]F-azomycin arabinoside 17 is controversial. Remarkably, while treatment with systemic agents is mandatory in case of high metastatic burden, patients with oligometastatic disease are the ideal candidates for alternative strategies, including initial surveillance 18 or surgery of the primary together with metastasectomy 19. Precise staging of the lymph nodes is even more problematic, since most clinically suspicious and enlarged nodes at standard imaging (cN1) are falsely positive (pT0) owing to the interaction between cancer and immune system 20 and trial specifically designed to test other radiopharmaceutical for the detection of lymph node involvement in renal cancer yielded negative results 17. Consequently, CAIX-PET might support surgeons at planning the most appropriate lymph node dissection template 21.

This report stands out for its novelty and uniqueness, nonetheless it is inherently limited by its pilot and explorative nature. Therefore, the current sample size prevents any aggregate quantitative or comparative analysis.

## Conclusions

Our findings generate the hypothesis that CAIX-PET might yield crucial information on cancer aggressiveness and systemic staging. Formal testing in clinical trials with adequate sample size and longitudinal evaluation of oncologic outcomes is needed to provide recommendations on the clinical benefit of CAIX-PET in high-risk renal cancer.

## Supplementary Material

Supplementary material, (Supplementary Table 1 and Supplementary Figures 1 and 2), accompanies this manuscript and forms an integral part of the submission.

Video 1.

## Figures and Tables

**Figure 2:**
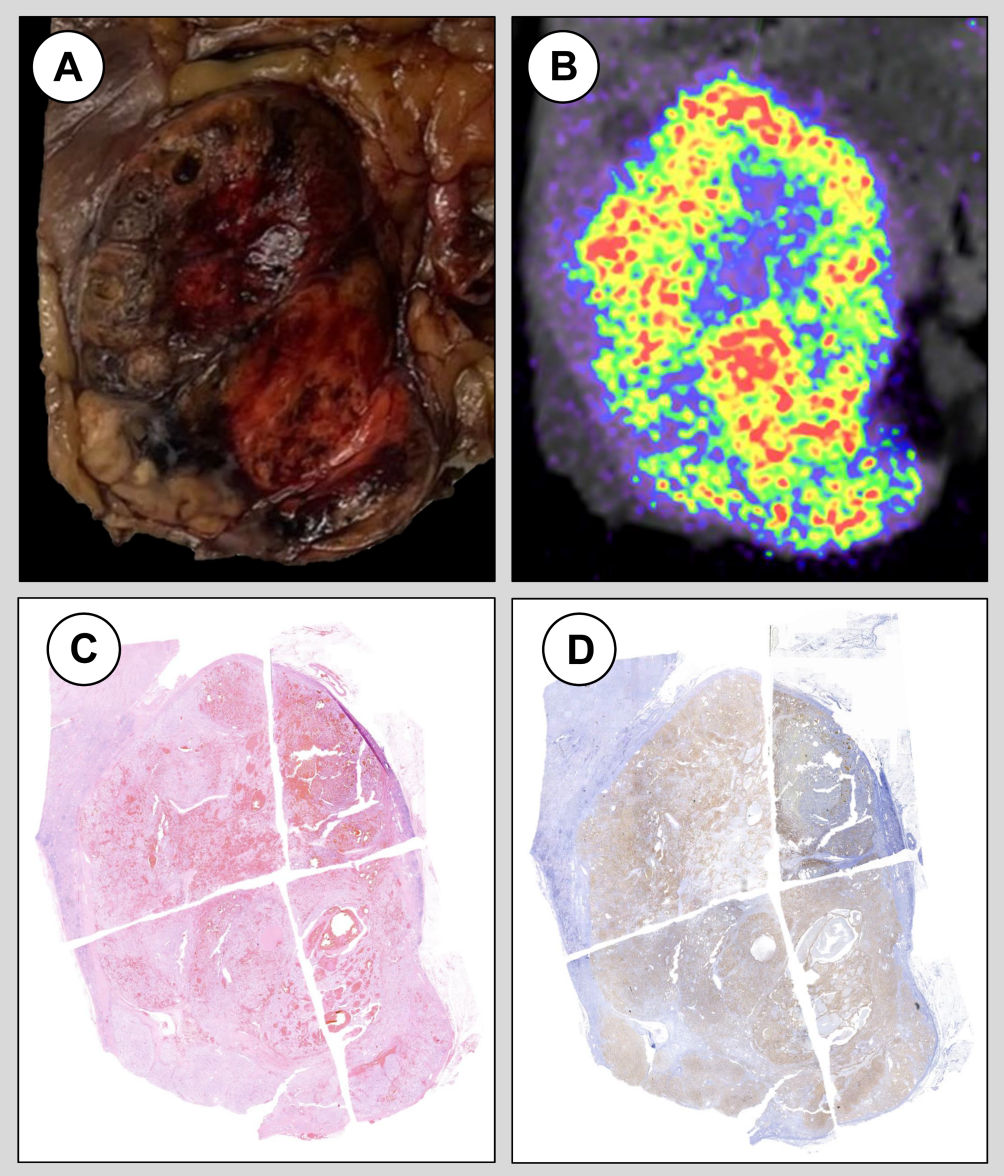
Macroscopic examination of the surgical specimen (A), *ex-vivo* imaging at preclinical PET/CT (B), hematoxylin and eosin staining (C) and immunohistochemistry staining for CAIX (D) in a patient diagnosed with a pT3a G3 clear cell renal cell carcinoma following robot-assisted radical nephrectomy.

**Table 1:** Patients’ characteristics.

VARIABLE		CASE 1	CASE 2	CASE 3
*General history*	Age	46	61	57
	Sex	M	M	M
	BMI	24	27	26
	Preoperative eGFR^(ml/min/BSA)^	59	96	68
*Conventional imaging*	Side	R	L	L
	Tumor size^(cm)^	16	6	10
	cT stage	cT3b	cT3a	cT2b
	cN stage	cN1	cN0	cN1
	cM stage	cM0	cM0	cM0
*CAIX-PET*	Primary tumor	Positive	Positive	Negative
	cN stage	cN0	cN0	cN0
	cM stage	cM0	cM1	cM0
*Surgery*	Intervention	Radical Nephrectomy	Radical Nephrectomy	Radical Nephrectomy
	Lymph-node dissection	Hilar and para-caval	No	Hilar and para-aortic
	Approach	Open surgery	Robotic surgery	Open surgery
	Intraoperative complications	Pleural lesion and pneumothorax	None	None
	Operative time^(min)^	217	174	196
*Hospital stay*	Post-operative complications	Fever and acute respiratory distress syndrome	None	None
	Clavien-Dindo classification	2	0	0
	Length of stay^(days)^	10	3	7
*Pathology*	Histology	Clear cell	Clear cell	Chromophobe
	Grade	4	3	3
	pT stage	pT3a	pT3a	pT3a
	pN stage	pN0	pNx	pN0
	pM stage	pMx	pM1	pMx
	Necrosis	Yes	No	Yes
	LVI	Yes	No	Yes
	Sinus fat invasion	Yes	Yes	Yes
	Perirenal fat invasion	No	No	No
	Renal vein invasion	No	No	No
	Adrenal gland	Not infiltrated	Not infiltrated	Not infiltrated
	Margins	R0	R0	R0

## Data Availability

The data generated in the current study are publicly available (DOI: 10.5281/zenodo.19050017).

## Abbreviations

CAIX: Carbonic Anhydrase IX
PET: Positron Emission Tomography
ccRCC: Clear Cell Renal Cell Carcinoma
Zr: Zirconium
MBq: Megabecquerel
mSv: Millisieverts
F: Fluorine
TC: Technetium
