# Supplementary material for: First-in-human ex-vivo validation of carbonic anhydrase IX-PET for high-risk renal cancer (CAT-VHL - PNRR-POC-2023-12377493)
Source: Theranostics. 2026 May 18;16(12):6980–7. doi: 10.7150/thno.122256 (PMC13232429; doi:10.7150/thno.122256)

Supplementary Table 1 - Scanner characteristics and acquisition protocol.

**Acquisition parameters**	**GE Healthcare Omni Legend**	
	PET	CT
**[89Zr]Zr-girentuximab**	37 MBq ± 10%	–
**Min/bed position**	5	–
**Crystal**	Digital BGO	–
**Reconstruction**	Qclear 1500	–
**DFOV (CM)**	70	-
**Matrix (pixels)**	384×384	512×512
**Resolution (mm)**	1.82X1.82	1.37X1.37
**Slice thickness (mm)**	2.07	2.5
**Slices**	963	943
**Voltage (kV)**	–	120
**Tube current (mA)**	–	150

Supplementary Figure 1 - The photomicrographs provide a detailed view of the lesion, which displays a rich vascular network and is composed of neoplastic cells with abundant clear cytoplasm, arranged in a complex tubular and nested architectural pattern. Importantly, no areas of necrosis were identified. At low magnification (100X; circles, left), the nuclei of the neoplastic cells already showed conspicuous eosinophilic nucleoli, which became more prominent and sharply delineated at higher magnification (200X; circles, right). These cytological and features are consistent with an overall grade 3, in accordance with the ISUP/WHO nucleolar grading system currently applied to renal neoplasms.

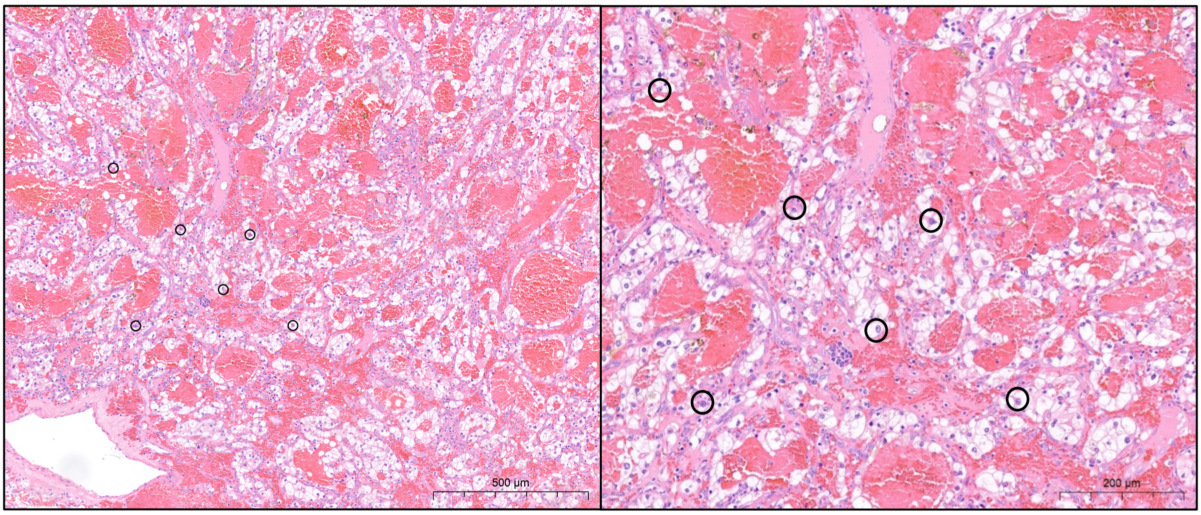

Supplementary Figure 2 - Descriptive analysis of the overlap between *ex-vivo* imaging at preclinical PET/CT and immunohistochemistry staining for CAIX by manual supoerinposition with increasing transparency showing spatial correspondence of regions with increased radiopharmaceutical uptake and regions with high expression of CAIX at immunohistochemistry.

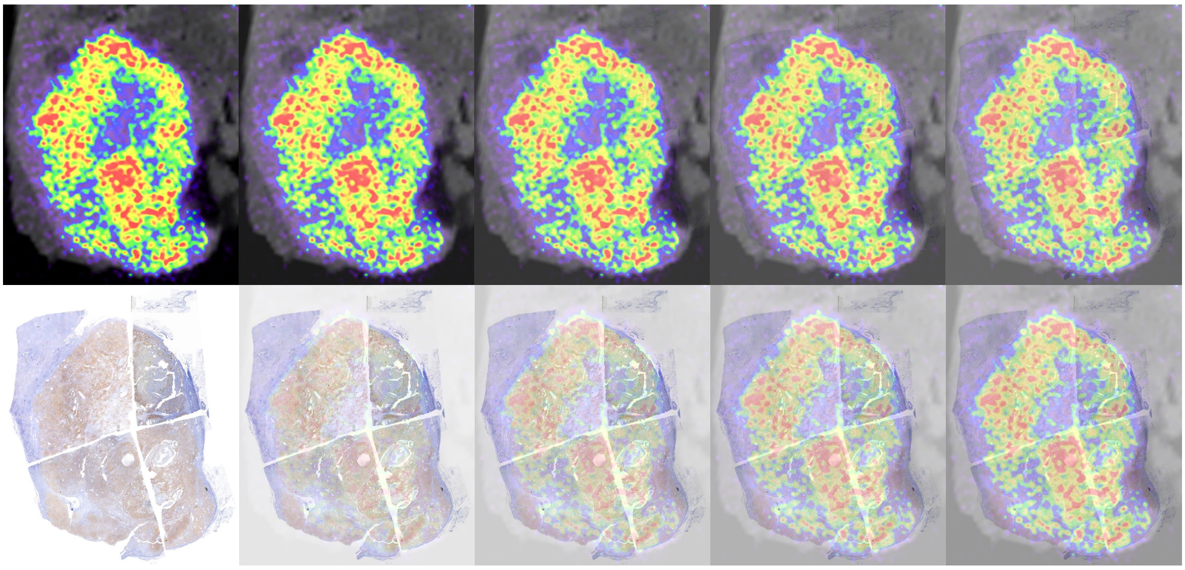

Supplement: Supplementary file 1 — Supplementary material, (Supplementary Table 1 and Supplementary Figures 1 and 2), accompanies this manuscript and forms an integral part of the submission. [file thnov16p6980s1.pdf]
